# The Tendency to Hæmorrhage in Jaundice and Allied Conditions

**Published:** 1907-06-01

**Authors:** 


### Points in Treatment.

### THE TENDENCY TO HÆMORRHAGE IN JAUNDICE AND ALLIED CONDITIONS.

### Prophylactic Administration of Calcium Chloride.

It is well known that a patient who is jaundiced has a greater tendency to bleed than has a healthy person. This is of importance both from a medical and from a surgical point of view. The following is a case which brings this home to one :—

A lady of 45 years of age had been suffering from attacks of severe colicky pain in the region of the gall-blader for some months; for eight weeks past there had been increasing jaundice; a fairly definite lump was felt beneath the tip of the right ninth rib, but the patient was too stout to allow of detailed palpation. She had lost flesh slightly, but not more than could be accounted for by the anxiety, pain, and loss of sleep and appetite. In short, it was clear that the condition was due either to gall-stones and inflammation, or to gall-stones plus growth. It was decided that an exploratory operation was advisable, and laparotomy was therefore performed. Only a small incision was made; extensive and inoperable malignant growth of the gall-bladder was found; nothing further was done, and the wound was sutured. Shortly after returning to bed the patient became collapsed and blanched; she lived a few hours longer, but died with all the symptoms of an extensive hæmorrhage. There was no oozing from the wound, nor any bleeding into the peritoneal cavity. At the autopsy there was over 20 feet of solid blood-clot entirely filling the small intestine. The autopsy revealed no bleeding-point, nor any ulcer of the stomach, duodenum, or bowel. The appearances suggested that there had been a general oozing from the mucosa of the intestine, attributable to the hæmorrhagic tendency produced by the cholæmia.

#### Hæmorrhage in Jaundice.

It is true that it is by no means in every case of jaundice that bleeding occurs so readily and to such a fatal extent. It is, however, difficult to pick out those cases in which hæmorrhage is going to occur from those in which there is little likelihood of it. Therefore operations in cases of jaundice should only be advised after the most careful and mature deliberation. The hæmorrhage described above was the result of very slight manipulation. Similar hæmorrhage from slight causes may readily occur without either anæsthetic or operation. Epistaxis, for example, is not at all uncommon, and it may be very severe. Hæmorrhage from the colon is also well known. Subcutaneous ecchymoses may result from comparatively gentle palpation. In a severe case of jaundice due to cirrhosis of the liver, after palpating the abdomen in the usual way in the endeavour to feel the liver and the spleen, it is not at all uncommon to find bruise-like marks appearing within a few hours at the sites of manipulation. These bruises may remain for a week or more and it is easy to see that in a medico-legal case they might be regarded as evidence of undue vigour, or even violence, in the treatment of the patient. Similar bruises may occur anywhere in such cases; the nurse or attendant, in merely lifting the patient in the ordinary way, may cause extensive ecchymoses and bruising of the arms and body.

#### The Medico-Legal Aspect.

Nor is it only when jaundice is actually present that this bruising readily occurs; it takes place in various hepatic affections even when the jaundice is quite slight. The importance of this may be very great from a medico-legal point of view. A patient suffering from cirrhosis of the liver may develop delirium tremens: friends or relatives may cause extensive bruising by a perfectly moderate restraint, and if death ensues suspicions of foul play may arise. Quite recently a case of this kind very nearly ended in a husband being sent to gaol; he would have been had not the doctor been exceedingly good at giving his evidence. In the end it was clearly proved that the man had been most forbearing and patient, driven almost to his wits' ends to know how to cure his wife, and never using any violence even when most provoked.

#### Some Practical Points.

We have dealt so far with hæmorrhages which have been, so to speak, spontaneous; that is to say, without actual gross severance of vessels. The condition is equally important in connection with bleeding from cut or broken surfaces. A tooth extraction, for example, may be followed by bleeding almost as difficult to stop as is that of hæmophilia. Incisions made by the surgeon need even more careful attention than usual, the minutest bleeding-points being closed to prevent subsequent oozing; in the case of actual vessels it is safer not to rely on torsion with forceps only, but to apply catgut or other ligatures.

#### How is the Bleeding Caused?

The question arises: How do jaundice and liver affections lead to this tendency to hæmorrhage? In the first place, neither the bile pigments nor the bile salts are the cause of the trouble; at least, both bile pigments and bile salts may be experimentally mixed with the circulating blood in large proportions without there being any immediate tendency to hæmorrhage. This is important, because it confirms the clinical experience that jaundice may be intense, without corresponding bleeding propensities: for example, in cases of simple catarrhal jaundice. Moreover, in some cases the tendency to bleed may be very great though the jaundice may be but slight. It seems that jaundice is more liable to lead to hæmorrhage when it has been long existent. The physician will consequently be loth to recommend any operation when his patient has been long jaundiced, and will advise treatment by calcium, described below, for some days before the operation is undertaken.

Secondly, there seems to be some alteration in the walls of the smaller vessels and capillaries; otherwise it is difficult to understand how general oozing into the intestine can come about, or subcutaneous ecchymosis from quite gentle palpation. We know of no medicines that will make the vessels strong again so long as the jaundice lasts.

### A Third View.

In the third place, there seems to be a change in the blood itself; experiments have shown that this is mainly one of deficient coagulative power. The coagulation time of the blood in many cases of severe and profound jaundice is very much prolonged. It has been found that this depends in no way upon deficiency in fibrinogen, for as much of this is present as in normal blood; the fault lies with the fibrin ferment (thrombokinase), which is formed very slowly in these patients. This is a very important idea, because it at once suggests a possible means of relieving this part of the danger. The influence of small quantities of calcium salts upon the rapidity of formation of fibrin ferment is well known; it is wise, therefore, to administer calcium to these patients in some soluble form. Calcium chloride may be given in 10-grain doses three times a day. It is true that hæmorrhage may still occur, notwithstanding the administration of calcium; but this line of treatment has certainly seemed to minimise the danger in many cases. It ought to be given, if possible, for several days or a week before the performance of a dental extraction or a surgical operation in such patients.

